# Co-expression of YAP and TAZ associates with chromosomal instability in human cholangiocarcinoma

**DOI:** 10.1186/s12885-021-08794-5

**Published:** 2021-10-06

**Authors:** Marcell Tóth, Lilija Wehling, Lena Thiess, Fabian Rose, Jennifer Schmitt, Sofia M. E. Weiler, Carsten Sticht, Carolina De La Torre, Melina Rausch, Thomas Albrecht, Niels Grabe, Lea Duwe, Jesper B. Andersen, Bruno C. Köhler, Christoph Springfeld, Arianeb Mehrabi, Yakup Kulu, Peter Schirmacher, Stephanie Roessler, Benjamin Goeppert, Kai Breuhahn

**Affiliations:** 1 Institute of Pathology, University Hospital Heidelberg, Im Neuenheimer Feld 224, 69120 Heidelberg, Germany; 2 Centre for Organismal Studies/BioQuant, Heidelberg University, Heidelberg, Germany; 3 NGS Core Facility, Medical Faculty Mannheim, Heidelberg University, Heidelberg, Germany; 4 Hamamatsu Tissue Imaging and Analysis Center (TIGA), BioQuant, Heidelberg University, Heidelberg, Germany; 5 Biotech Research and Innovation Centre (BRIC), Department of Health and Medical Sciences, University of Copenhagen, Copenhagen, Denmark; 6 Department of Medical Oncology, National Center for Tumor Diseases, University Hospital Heidelberg, Heidelberg, Germany;, Liver Cancer Center Heidelberg, University Hospital Heidelberg, Heidelberg, Germany; 7 Department of General Visceral and Transplantation Surgery, University Hospital Heidelberg, Heidelberg, Germany

**Keywords:** Hippo pathway, CIN25, Liver cancer, Cell density, Genomic instability

## Abstract

**Background:**

Activation of the oncogene *yes-associated protein* (YAP) is frequently detected in *intrahepatic cholangiocarcinoma* (iCCA); however, the expression pattern and the functional impact of its paralogue *WW domain-containing transcription regulator 1* (WWTR1; synonym: TAZ) are not well described in different CCA subtypes.

**Methods:**

Immunohistochemical analysis of YAP and TAZ in iCCA and *extrahepatic CCA* (eCCA) cohorts was performed. YAP/TAZ shuttling and their functional impact on CCA cell lines were investigated. Target genes expression after combined YAP/TAZ inhibition was analyzed.

**Results:**

Immunohistochemical analysis of iCCA and eCCA revealed YAP or TAZ positivity in up to 49.2%; however, oncogene co-expression was less frequent (up to 23%). In contrast, both proteins were jointly detectable in most CCA cell lines and showed nuclear/cytoplasmic shuttling in a cell density-dependent manner. Next to the pro-proliferative function of YAP/TAZ, both transcriptional co-activators cooperated in the regulation of a gene signature that indicated the presence of *chromosomal instability* (CIN). A correlation between YAP and the CIN marker *phospho-H2A histone family member X* (pH2AX) was particularly observed in tissues from iCCA and *distal CCA* (dCCA). The presence of the CIN genes in about 25% of iCCA was statistically associated with worse prognosis.

**Conclusions:**

YAP and TAZ activation is not uncoupled from cell density in CCA cells and both factors cooperatively contribute to proliferation and expression of CIN-associated genes. The corresponding group of CCA patients is characterized by CIN and may benefit from YAP/TAZ-directed therapies.

**Supplementary Information:**

The online version contains supplementary material available at 10.1186/s12885-021-08794-5.

## Background

Together with the transcriptional co-activators *yes-associated protein* (YAP) and *WW domain containing transcription regulator 1* (WWTR1; synonym: TAZ), the Hippo signaling pathway is a key regulator of organ size under physiological conditions [1]. Controlled by cell-cell contact, cell density and cell polarity, inactivation of a central kinase cassette consisting of *mammalian STE20-like protein kinase* (MST)-1/2 and *large tumor suppressor kinase* (LATS)-1/2 is leading to YAP/TAZ hypophosphorylation and nuclear translocation followed by their interaction with transcription factors such as *TEA domain transcription factors* (TEADs) family members or *forkhead box M1* (FOXM1) [2, 3].

Dysregulation of the Hippo/YAP/TAZ signaling axis has been described for many solid tumors of the gastrointestinal tract such as pancreatic cancer, colon cancer and the most common primary liver cancer, hepatocellular carcinoma (HCC) [4]. As shown for HCC, inactivation of the Hippo kinase cassette or activation of YAP is leading to liver overgrowth via expansion of liver progenitor cells and eventually cancer development with combined hepatocellular/cholangiocellular differentiation [5]. Mechanistically, the complex consisting of YAP and transcription factors promotes the expression of genes involved in DNA replication, cell cycle regulation, chromosomal segregation, but also in the control of cellular stemness. In this context, YAP-dependent proliferation of liver cells is leading to the accumulation of mutations and the induction of chromosomal instability (CIN) [3]. However, the oncogenic role of TAZ in hepatocarcinogenesis is less defined, although previous results illustrated both tumor-initiating and tumor-supporting properties [6, 7]. In addition, recent data demonstrated that TAZ cooperates with YAP in HCC progression [7].

For cholangiocarcinoma (CCA), the second most common primary liver cancer, overexpression of YAP was described in 32 to 98% of patients [8–12]. This surprisingly wide range of YAP positivity in independent CCA cohorts might be due to different scoring algorithms (e.g., if only nuclear and/or cytoplasmic YAP positivity was considered) or variable cohort composition with regard to CCA subtypes. In this context, a precise analysis of YAP expression discriminating between *intrahepatic cholangiocarcinoma* (iCCA) and *extrahepatic cholangiocarcinoma* (eCCA) is missing so far. In addition, a detailed analysis of the YAP paralogue TAZ in CCA subgroups and its potential tumor-supporting function in combination with YAP has not been considered yet. The last point is of special importance for two reasons: a. several studies for different tumor entities illustrated that YAP and TAZ partly cooperate in the regulation of tumor-supporting functions [7, 13]. b. intensive pharmaceutical research is ongoing to develop drugs that block both YAP and TAZ activity [14, 15].

In this study, a systematic characterization of YAP and TAZ expression in iCCA and eCCA, the latter consists of perihilar and distal cholangiocarcinoma (pCCA and dCCA, respectively), revealed mutually exclusive nuclear enrichment of YAP and TAZ in about half of all cases. Although nuclear co-expression of both proteins was observed only in the minority of CCAs, YAP and TAZ were cooperatively expressed in most CCA cell lines, supporting cell proliferation and expression of factors involved in CIN. The association between YAP, CIN gene signature and poor clinical outcome was confirmed in human CCA patient cohorts.

## Methods

Primers and antibodies used in this study is provided as Supplementary Table S2.

### Patient cohort and tissue microarray (TMA)

Tissue samples organized on three TMAs and clinical data derived from 152 iCCA patients, 155 pCCA patients and 126 dCCA patients were used for this study as previously described (Supplementary Table S1) [16]. In brief, the iCCA TMA contained 152 different carcinomas with well to poor differentiation (grading: G1 = 9, G2 = 98, G3 = 42, G4 = 3). Due to the low number of G4 tumors, results from G3 and G4 tumors were merged for the statistical analysis. The pCCA TMA included 155 tumor tissues (grading: G1 = 8, G2 = 114, G3 = 33, G4 = 0), while the dCCA TMA consisted of 126 tumor samples (G1 = 1, G2 = 84, G3 = 41, G4 = 0). For each patient two independent tissue cores were analyzed.

For semiquantitative data analysis of staining intensities, an evaluation score was calculated based on the following scoring system: quantity (0 = no expression; 1 = less than 1% positive cells; 2 = 1–9% positive cells; 3 = 10–50% and 4 = more than 50% positive cells) and intensity (0 = no expression; 1 = low expression; 2 = medium expression; 3 = strong expression). Subsequently, the product of quantity and intensity was calculated for each dot (range: 0–12). Finally, the arithmetic mean value of the two available samples/dots for each tumor was calculated. The obtained IHC scores for each staining were correlated with each other and with tumor grade using Spearman’s rank correlation. For presentation, protein positivity was classified into low/no expression (mean values from 0 to 3), intermediate positivity (mean values from 3.5–7.5) and high positivity (mean values from 8 to 12). For YAP and TAZ, the cytoplasmic and nuclear staining scores were evaluated separately.

### CCA transcriptomic data

Published gene expression data from 104 CCA patients and 6 healthy donors was used to analyze the expression of the CIN25 gene signature (GSE26566) [3, 17, 18]. Data was normalized by applying the CPM method, clustered into 3 groups by k-mean clustering algorithm and the differential expression was visualized as heatmap using the R package ComplexHeatmap [19]. A signature score was calculated by scaling the expression of each gene across all patient data followed by adding up all individual gene values for each patient. Therefore, the expression of each gene contributed equally to the calculated signature score.

### Immunohistochemical staining protocol

TMAs were cut in 1–2 μm thick sections using a microtome. Tissues were deparaffinized in xylol for 3 × 5 min. Afterwards, a rehydration process was performed using an ethanol gradient (2 × 2 min of 100% ethanol, 96% ethanol and 70% ethanol), followed by a washing step with distilled water. For antigen retrieval, the slides were pre-treated with Dako Target Retrieval Solution pH 6.0 (MCM2, YAP, TAZ and pH2AX) in a steam cooker for 8 (YAP), 15 (MCM2) or 30 (TAZ, pH2AX) minutes. After cooling down and rinsing in distilled water, the sections were placed in TBST for 10 min. The primary antibodies were diluted in antibody diluting buffer (Dako, Hamburg, Germany) and incubated in a wet chamber at room temperature for 1 h (YAP, TAZ) or at 4 °C overnight (MCM2, pH2AX). This was followed by a washing cycle with TBST for 2 × 5 min.

For MCM2, alkaline phosphatase-based Detection Line SuperVision Red 2 AP system (DCS, Hamburg, Germany) was used. The remaining tissue sections were incubated with rabbit Enhancer Detection Line for 20 min, washed with TBS two times (2 × 5 min) and incubated with AP-Polymer Detection Line for 20 min. After additional TBS washing (2 × 5 min), the signal development was done with Liquid Permanent Red (Zytomed Systems, Berlin, Germany) for 23 min.

For signal detection of TAZ- and YAP-immunohistochemistry, POLYVIEW® PLUS AP anti-rabbit reagent (Enzo Life Sciences, Lörrach, Germany) was used for 1 h. This was followed by two washing steps with TBST (2 × 5 min) and 8–15 min incubation period with Liquid Permanent Red.

For pH2AX stain, the Streptavidin horseradish peroxidase (HRP) detection system was used (Dako). Samples were incubated with Dako REAL biotinylated secondary antibodies for 25 min, washed with TBST two times (2 × 5 min) and blocked with Dako REAL Peroxidase-Blocking Solution for 10 min. The tissue sections were washed again with TBST (2 × 5 min) and incubated with Streptavidin-HRP for 20 min. After the last washing step (2 × 5 min with TBST), the signal development was achieved with 3-amino-9-ethylcarbazole (AEC; Dako) for 7 min. The above detailed immunohistochemical stains were carried out by the IHC research facility at the Institute of Pathology (Heidelberg).

The Ki-67 staining was carried out on the VENTANA automated slide stainer following the manufacturer’s protocol in the NCT-Biobank Heidelberg (Dako).

A list of antibodies used in this study is provided as Supplementary Table S2.

### Cell culture and genetic RNAinterference (RNAi)

The human CCA cell lines HUCCT-1 (JCRB0425), HuH-28 (JCRB0426) and NOZ (JCRB1033) were purchased from *Japanese Collection of Research Biosources* (JCRB). The cell line G415 (RCB2640) was purchased from the RIKEN BioResource Research Center. Cells were cultured in RPMI (Sigma Aldrich, Steinheim, Germany) or DMEM (Gibco/Thermo Fisher Scientific, Waltham USA) containing 10% fetal bovine serum and 1% penicillin/streptomycin at 37 °C in a 5% CO_2_ atmosphere. Cells were routinely tested for mycoplasma contamination and authentication was performed by STR analysis (DSMZ, Braunschweig, Germany).

Gene-specific small interfering RNAs (siRNAs) were obtained from Eurofins MWG Operon (Ebersberg, Germany). For siRNA inhibition assays, cells were directly transfected after trypsinization. Scrambled siRNA-treated cells served as control. Gene-specific siRNAs (final concentration of 40 nM for combinations of two siRNAs for YAP and TAZ) and the transfection reagent Lipofectamine RNAiMAX (Gibco/Thermo Fisher Scientific) were diluted, mixed in Opti-MEM (Gibco/Thermo Fisher Scientific) and incubated at room temperature, before being distributed onto the cells. Twenty-four hours after transfection, medium was replaced and cells were either reseeded for functional analyses or harvested after 48 h. The following siRNAs were used: scrambled siRNA (UGG UUU ACA UGU CGA CUA A-dT-dT), YAP siRNA #1 (CCA CCA AGC UAG AUA AAG A-dT-dT), YAP siRNA #2 (GGU CAG AGA UAC UUC UUA A-dT-dT), TAZ siRNA #1 (AAA CGU UGA CUU AGG AAC UUU-dT-dT), and TAZ siRNA #2 (AGG UAC UUC CUC AAU CAC A-dT-dT).

### Quantitative PCR (qPCR) and Western blotting

Total RNA was isolated using the NucleoSpin RNA II kit (Macherey-Nagel, Düren, Germany) and reverse transcription was performed using 500 or 1000 ng of total RNA (Revert Aid H Minus RT, Thermo Fisher Scientific). qPCR reactions were set up using the primaQuant 2x qPCR-CYBR-Green-Mastermix (Steinbrenner Laborsysteme, Wiesenbach, Germany) with the following cycling conditions: 95 °C for 15 min, followed by 40 cycles of 95 °C for 15 s, and 60 °C for 60 s. Product specificity was confirmed by melting curve analysis (95 °C for 15 s, 60 °C for 30 s, 60–95 °C 0.5 °C/second). β2-microglobulin (B2M) or ribosomal protein L41 (RPL41) were used for normalization. A list of primers used in this study is provided as Supplementary Table S2.

Total protein extracts were isolated using 10x Cell Lysis Buffer (Cell Signaling/New England Biolabs, Frankfurt, Germany) and separated using 8 to 10% sodium dodecyl sulfate-polyacrylamide gel electrophoresis (SDS-PAGE) and electro-transferred to a nitrocellulose membrane. After blocking in 5% milk or BSA in TBST, primary antibodies were added and the membrane was incubated at 4 °C overnight. The appropriate secondary antibodies (1:20,000; IRDye 680 and 800, LiCor Biosciences, Bad Homburg, Germany) were diluted in milk or BSA in TBST. All results from Western Immunoblotting were confirmed by independent experiments. Signal detection was done using the Odyssey-SA system (LiCor Biosciences).

### Immunofluorescence (IF) and quantitative measurement of YAP and TAZ localization

Cells were seeded on Poly-L-Lysine coated glass slides under different cell density conditions from very low (30,000 in 12 well plate) to very high density (550,000 in 12 well plate) 1 day prior staining. Fixation was done using paraformaldehyde (4% PFA in PBS; 15 min) followed by permeabilization (0.2% Triton X-100, 7 min). Cells were blocked (1% BSA in PBS) and treated with primary (rabbit anti-YAP, 1:60; rabbit anti-TAZ, 1:25) and Alexa-488 conjugated secondary antibodies (donkey anti-rabbit, 1:300) followed by DAPI staining.

Imaging in two channels (DAPI λ = 409.1 nm; Alexa 488 λ = 483.7 nm) was performed using a Nikon A1 plus laser scanning confocal microscope (Nikon Imaging Center at the University of Heidelberg, BioQuant). A Nikon Plan Apo λ 20x NA 0.75 objective (WD 1 mm, FOV 0.64 × 0.64 mm, refractive index 1.00) with integrated PFS was used. In total, 238 images (G415), 120 images (HUCCT-1), 514 images (HuH-28) and 228 images (NOZ) were obtained. Subcellular localization of YAP and TAZ was analyzed in an automatic manner using Fiji image processing software. For this, nuclei and cytoplasm were segmented using Weka Segmentation algorithm; on the resulting probability maps a threshold was applied and afterwards nuclear areas were subtracted from the cytoplasmic areas [20, 21]. Resulting masks were overlaid with the original images and the intensities within cytoplasm and nucleus were obtained. Subsequently, the mean nuclear and cytoplasmic intensities within the field of view/image were divided and the nuclear to cytoplasmic ratios were obtained.

### Viability assay and BrdU enzyme-linked immunosorbent assay (ELISA)

For measuring viability, CCA cells were seeded on a 96-well plate 24 h after siRNA transfection. After further 48 and 72 h, Resazurin reagent (R&D Systems, Minneapolis, USA) was added according to the manufacturer’s protocol.

For analyzing proliferation after gene-specific inhibition, sub-confluent CCA cells were analyzed using a BrdU-ELISA assay (Cell proliferation ELISA Biotrak, GE Healthcare/Amersham, Freiburg, Germany) 48 h after transfection. Relative proliferation is shown as absorbance normalized against scrambled siRNA-treated cells.

### Expression profiling

For the analysis of TAZ- and/or YAP-regulated genes, HUCCT-1 cells were transfected with TAZ and YAP-specific siRNAs (40 nM; siRNA TAZ #2 and siRNA YAP #1). Total RNA was harvested 24 h after transfection. Prior to hybridization, RNA integrity was measured (Agilent 2100 Bioanalyzer, Agilent, Frankfurt, Germany). Only samples with an RNA integrity number (RIN) > 7 were used for transcriptomics. Purified and fragmented complementary RNA was generated according to the manufacturer’s instructions using the GeneChip® WT Plus Reagent Kit. Fragments were biotin-labelled prior to hybridization on Clariom D human chips using a GeneChip Hybridisation oven 640 (Thermo Fisher Scientific). Successive staining and scanning were performed with a GeneChipFluidics Station 450 and a GeneChip Scanner 3000, respectively (Thermo Fisher Scientific).

After gene annotation using a custom CDF file version 22, the fluorescence intensity was measured, RMA background-corrected, normalized, and differential expression was statistically assessed using the software package SAS JMP7 genomics (SAS Institute, Cary, USA). As cut-off, a *false discovery rate* (FDR) value of 0.05 was considered as significant. To assure a homogeneous distribution of the generated data, *principal component analysis* (PCA) was performed to compare the similarity of individual biological samples in this study. To identify pathways and cellular processes with significant enrichment of differentially expressed genes, *gene set enrichment analysis* (GSEA) was performed. To describe expression differences, relative values were displayed by annotating blue and red to the minimum and maximum intensity for each gene. Raw and normalized data were deposited in the Gene Expression Omnibus database (available: http://www.ncbi.nlm.nih.gov/geo/; accession number: GSE172135).

### Software and statistics

Data are presented as mean ± standard deviation. The Spearman‘s rank correlation coefficient (r) was used as a statistical measure of association. Overall survival was analyzed by the Kaplan-Meyer method using the Log-rank (Mantel-Cox) test. Statistical comparison between two groups was performed using the non-parametric Mann-Whitney U test. All statistical analyses were performed using Prism 8 (GraphPad Prism Software, San Diego, USA) and SPSS (IBM Corp, Armonk, NY, USA). Significance levels were defined as: p* ≤ 0.05, p** ≤ 0.01, and p*** ≤ 0.001. The Morpheus online tool was used for the visualization of array data and generation of heatmaps (https://software.broadinstitute.org/morpheus/).

## Results

### YAP and TAZ protein expression in human iCCA and eCCA tissues

**Fig. 1:**
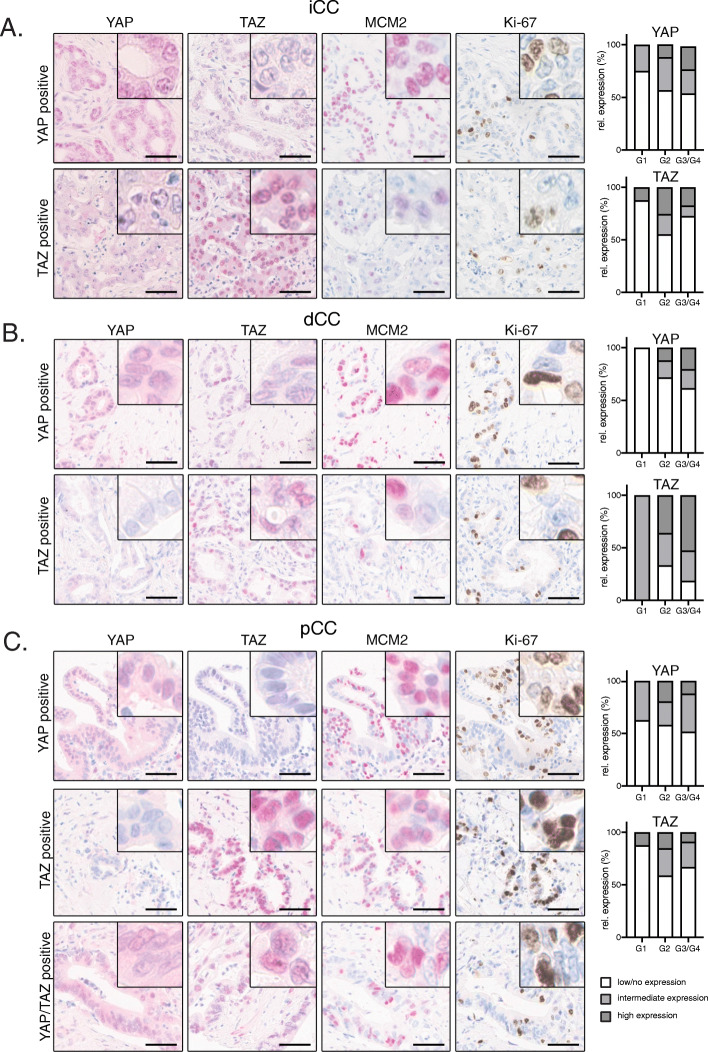
YAP and TAZ expression in human iCCA, pCCA and dCCA. Representative IHC stains for YAP, TAZ, MCM2 and Ki-67 in cohorts of CCA subgroups of iCCA (**A**, *n* = 152), dCCA (**B**, *n* = 126) and pCCA (**C**, *n* = 155). For each subtype, a representative YAP and TAZ-positive example is shown. For pCCA an additional case with nuclear YAP/TAZ is shown. Bars: 50 μm. Adjacent bar graphs illustrate the relative nuclear positivity of YAP or TAZ for the respective CCA subgroup with regard to tumor grading (white: low/no expression; light grey: intermediate expression; dark grey: high expression)

To obtain a comprehensive and comparative overview of YAP and TAZ expression in iCCA and eCCA (with pCCA and dCCA), tissue microarrays were designed consisting of 152 iCCA, 155 pCCA and 126 dCCA samples (Supplementary Table S1) [16]. Immunohistochemical stains for YAP and TAZ were followed by a careful histological evaluation of nuclear and cytoplasmic YAP/TAZ positivity (Fig. 1a-c).

For the individual CCA types, we detected a specific nuclear YAP positivity in 39.5% (iCCA), 41.9% (pCCA), and 29.4% (dCCA), while we observed nuclear TAZ for 31.6% (iCCA), 35.5% (pCCA) and 65.9% (dCCA) of all cases (Table 1). In total, 43.4% (iCCA), 32.3% (pCCA), and 49.2% (dCCA) showed nuclear staining for YAP or TAZ. Interestingly, a smaller percentage of tumors exhibited combined nuclear positivity for both factors (13.8% (iCCA), 22.6% (pCCA), and 23% (dCCA)).

**Table 1:** Distribution of nuclear and cytoplasmic YAP and TAZ positivity in human CCA subtypes

**iCC (152)**	**YAP**	**TAZ**	**YAP or TAZ**	**YAP and TAZ**
nuclear	60 (39.5%)	48 (31.6%)	66 (43.4%)	21 (13.8%)
cytoplasmic	43 (28.3%)	4 (2.6%)	43 (28.3%)	2 (1.3%)
nuc. and cytopl.	68 (44.7%)	49 (32.2%)	69 (45.4%)	24 (15.8%)
**pCC (155)**	**YAP**	**TAZ**	**YAP or TAZ**	**YAP and TAZ**
nuclear	65 (41.9%)	55 (35.5%)	50 (32.3%)	35 (22.6%)
cytoplasmic	48 (31%)	3 (1.9%)	49 (31.6%)	1 (0.6%)
nuc. and cytopl.	76 (49%)	57 (36.8%)	57 (36.8%)	38 (24.5%)
**dCC (126)**	**YAP**	**TAZ**	**YAP or TAZ**	**YAP and TAZ**
nuclear	37 (29.4%)	83 (65.9%)	62 (49.2%)	29 (23%)
cytoplasmic	39 (31%)	6 (4.8%)	43 (34.1%)	1 (0.8%)
nuc. and cytopl.	55 (43.7%)	84 (66.7%)	55 (43.7%)	42 (33.3%)

In addition to nuclear positivity, most CCAs also showed a prominent cytoplasmic staining for YAP and TAZ. Considering both nuclear and cytoplasmic staining, YAP or TAZ positivity was observed in 45.4% of iCCA, 36.8% of pCCA, and 43.7% of dCCA. In summary, we detected a prominent expression of YAP or TAZ in all CCA subtypes; however, nuclear co-accumulation of YAP and TAZ was considerably less frequent compared to individual YAP or TAZ protein expression in the nucleus or cytoplasm. These results were surprising for these two factors that are controlled by identical upstream mechanisms.

Although, a moderate increase of nuclear YAP/TAZ expression was seen upon tumor progression, no statistically significant association with tumor dedifferentiation was observed (Fig. 1a-c). No other associations between YAP/TAZ positivity and clinical parameters were detected (e.g., UICCA stage, sex, age and patient survival).

However, in all three CCA subtypes, nuclear YAP significantly correlated with the expression of the YAP target gene *minichromosome maintenance complex component 2* (MCM2; iCCA: *r* = 0.23, pCCA: *r* = 0.2, dCCA: *r* = 0.31; all *p* ≤ 0.01). In addition, nuclear YAP positivity correlated with the expression of the proliferation marker Ki-67 (iCCA: *r* = 0.42, pCCA: *r* = 0.33, dCCA: *r* = 0.30; all *p* ≤ 0.01). Interestingly, nuclear TAZ only correlated with nuclear YAP in the group of pCCA but not in iCCA or dCCA (pCCA: *r* = 0.43; *p* ≤ 0.001). Accordingly, TAZ was also not statistically associated with MCM2 and Ki-67 expression in iCCA and dCCA.

In summary, these data demonstrate that YAP and TAZ are expressed in all subtypes of human CCAs with comparable frequencies. Although controlled by the same upstream kinase cassette, nuclear YAP/TAZ co-accumulation is detected in a minority of CCA.

### Co-expressed YAP and TAZ dynamically respond to cell density in CCA cells

**Fig. 2:**
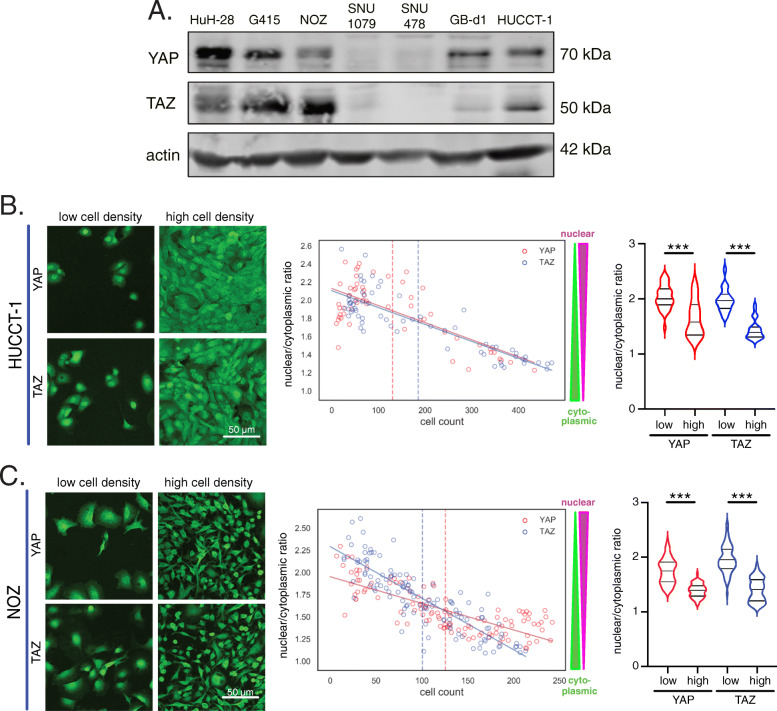
YAP and TAZ shuttle dynamically in cholangiocyte-derived cancer cells. **A** Detection of YAP and TAZ in total protein lysates from different cholangiocyte-derived cancer cell lines. iCCA cells (HuH-28, SNU1079, HUCCT-1), eCCA cells (SNU478) and gallbladder carcinoma cells (G415, NOZ, GB-d1) were tested. Actin served as loading control. HUCCT-1 (**B**) and NOZ (**C**) cells were cultured under variable cell density conditions. Exemplary immunofluorescence images with YAP and TAZ in cells for low- and high-density conditions are shown. Graphs illustrate the nuclear/cytoplasmic (n/c) ratio for YAP (red) and TAZ (blue). Higher n/c ratios indicate nuclear enrichment, while low ratios represent nuclear exclusion. Each dot represents one image. The dashed line defines the mean cell count over all fields of vision of the cell line. Values smaller than this threshold define low cell density and values above this threshold indicate high cell density. Violin plots summarize the results of all obtained images. Statistical test: Mann-Whitney U. Lines represent linear regression for YAP (red) and TAZ (blue)

To perform in vitro studies on the role of YAP and TAZ, different cholangiocyte-derived cancer cell lines were analyzed regarding their YAP and TAZ protein expression (Fig. 2a). For this, iCCA (HuH-28, SNU1079, HUCCT-1), eCCA (SNU478) and gallbladder carcinoma (G415, NOZ, GB-d1) cell lines were tested for their YAP and TAZ expression. According to the Western immunoblot results, YAP and TAZ showed a prominent co-expression in HuH-28, G415, NOZ, HUCCT-1 and, although with a less pronounced TAZ-expression, also in GB-d1 cells. In contrast, SNU1079 and SNU478 cells were negative for both factors. This was an interesting observation, because in our tissue analysis only a minority of CCA showed YAP and TAZ co-expression (Fig. 1). For this reason, we hypothesized that the presence of both factors may provide a growth advantage to some tumor cell lines (e.g., HuH-28, G415, NOZ, HUCCT-1), while for other cell lines YAP/TAZ-independent mechanisms may contribute to the cell-context-independent growth (e.g., SNU1079, SNU478).

In non-malignant cells and many tumor cells, activation of the Hippo pathway and subcellular localization of YAP/TAZ are regulated through cell-cell contact and matrix stiffness [22]. However, previous reports pointed to cell density-independent activation of YAP in CCA cells [8]. To comparatively investigate whether cholangiocyte-derived tumor cells from inside and outside the liver parenchyma responded to exogenous stimuli such as cell density, different cell lines with YAP/TAZ expression were investigated.

For this purpose, iCCA cells (HuH-28, HUCCT-1) and gallbladder cancer cells (G415, NOZ) with YAP and TAZ co-expression were cultured under low to high cell density conditions, followed by IF-based subcellular detection of YAP or TAZ. Nuclear and cytoplasmic YAP/TAZ levels were quantitatively measured for 120–514 arbitrarily taken pictures in an unbiased manner using a computational image analysis algorithm [20, 21]. The data revealed a clear cell density-dependent subcellular shuttling of YAP and TAZ in all analyzed cells; however, the cell lines showed different dynamics (Fig. 2b/c; Supplementary Figure S1A/S1B). While for HUCCT-1 cells both proteins were consistently regulated (low density: nuclear; high density: cytoplasmic; Fig. 2b), G415 cells demonstrated a more dynamic response for YAP, while TAZ translocation was less pronounced (lower slope of regression curve; Supplementary Figure S1B). Vice versa, in NOZ cells TAZ responded stronger to cell density than YAP (Fig. 2c).

Together, these results illustrate that cholangiocyte-derived cancer cell lines with YAP and TAZ co-expression are sensitive towards extracellular information and that the activation of YAP/TAZ is not uncoupled from cell density regulation. Cell line-specific molecular features may account for the observed differences of YAP and TAZ shuttling.

### YAP/TAZ supports viability and proliferation in iCCA and eCCA cells

**Fig. 3:**
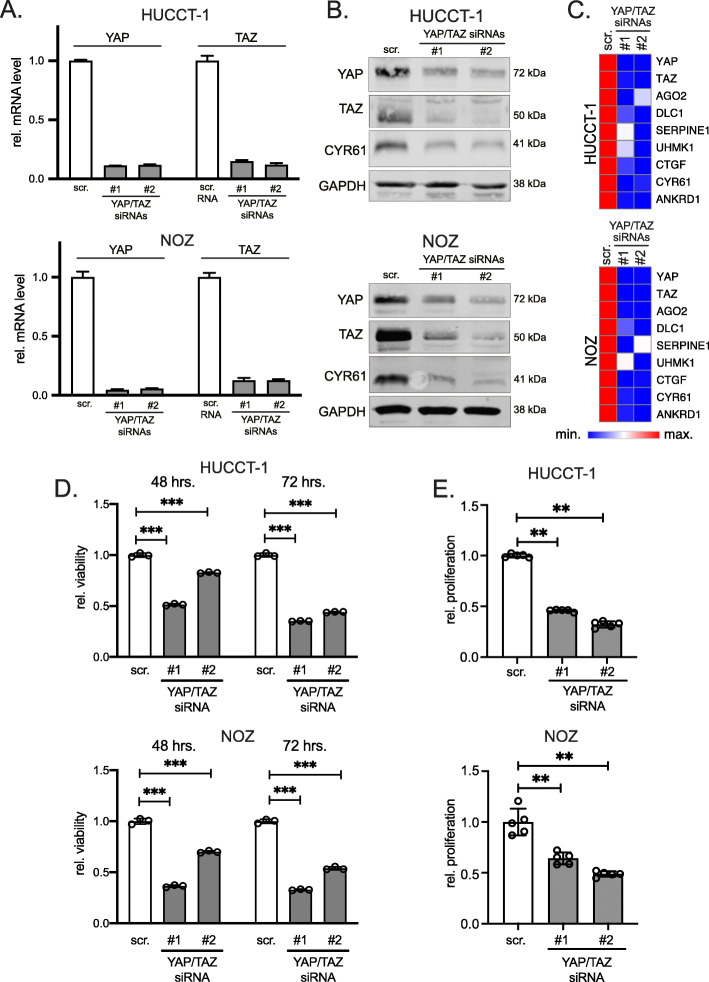
YAP/TAZ transcriptionally regulate and support cholangiocyte-derived cancer cell proliferation. **A** Real time PCR for YAP and TAZ in HUCCT-1 and NOZ cells after combined siRNA-mediated silencing of YAP and TAZ for 24 h. **B** Western immunoblotting and detection of YAP, TAZ and CYR61 in total protein lysates (HUCCT-1 and NOZ) 24 h after transfection of YAP/TAZ-specific siRNAs. GAPDH served as loading control. **C** Heatmaps summarize the results of real-time PCRs after combined YAP/TAZ silencing in HUCCT-1 and NOZ cells for 24 h. Known YAP/TAZ target genes were analyzed. **D** HUCCT-1 and NOZ cell viability assays were measured 48 and 72 h after transient transfection of YAP/TAZ-specific siRNA combinations. **E** Cell proliferation of HUCCT-1 and NOZ cells was measured 48 h after combined YAP/TAZ silencing. Scr. - scrambled siRNA (control). Statistical test: Mann-Whitney U

To systematically analyze the functional relevance of combined YAP and TAZ expression in iCCA and gallbladder cancer cells, the expression of both proteins was inhibited by RNAi. For this, we transiently transfected two independent siRNA combinations (siRNAs #1 and #2, respectively) for YAP and TAZ each to achieve a simultaneous silencing of both factors. Real-time PCR and Western immunoblotting corroborated the efficient inhibition of YAP and TAZ at mRNA and protein levels in all cell lines (Fig. 3a/b, Supplementary Figure S2A/S2B).

To confirm the transcriptional activity of YAP and TAZ, real-time PCR analysis after YAP/TAZ silencing was performed for seven known target genes *argonaute 2* (AGO2), *deleted in liver cancer 1* (DLC1), *plasminogen activator inhibitor 1* (PAI1/SERPINE1), *U2AF homology motif kinase 1* (UHMK1), *connective tissue growth factor* (CTGF), *cysteine-rich heparin-binding protein 61* (CYR61) and *ankyrin repeat domain 1* (ANKRD1), [3, 23]. As expected from the reduction of positive transcriptional regulators, combined YAP/TAZ silencing for 24 h reduced all tested factors (Fig. 3c, Supplementary Figure S2C).

To define the functional relevance of YAP and TAZ, we examined cell viability 48 and 72 h after the combined inhibition. For all tested cells, reduction of YAP/TAZ expression significantly diminished viability; however, only moderate effects were detectable for HuH-28 cells (Fig. 3d, Supplementary Figure S2D). For this reason, additional BrdU proliferation assays were performed to test for the relevance of YAP/TAZ overexpression on iCCA and gallbladder cancer cell mitosis. Indeed, YAP/TAZ silencing significantly reduced proliferation of all tested cells including HuH-28 cells (Fig. 3e, Supplementary Figure S2E). Interestingly, the weakest effect on viability and proliferation were observed for HuH-28, while for the other cell lines comparably stronger reduction was detected.

In sum, like for most other solid tumor types, YAP and TAZ control the expression of known target genes and support proliferation in different types of cholangiocyte-derived cancer cells.

### YAP and TAZ contribute to the expression of CIN-associated genes in cultured cholangiocyte-derived cancer cells

To further define the molecular mechanisms of how the Hippo pathway and its downstream effectors contribute to CCA development, expression profiling of HUCCT-1 cells after YAP/TAZ silencing was performed. For this, total mRNA was isolated 24 h after siRNA transfection and subjected to gene expression analysis (scrambled control siRNA vs. YAP/TAZ siRNAs; *n* = 3 for each biological condition).

**Fig. 4:**
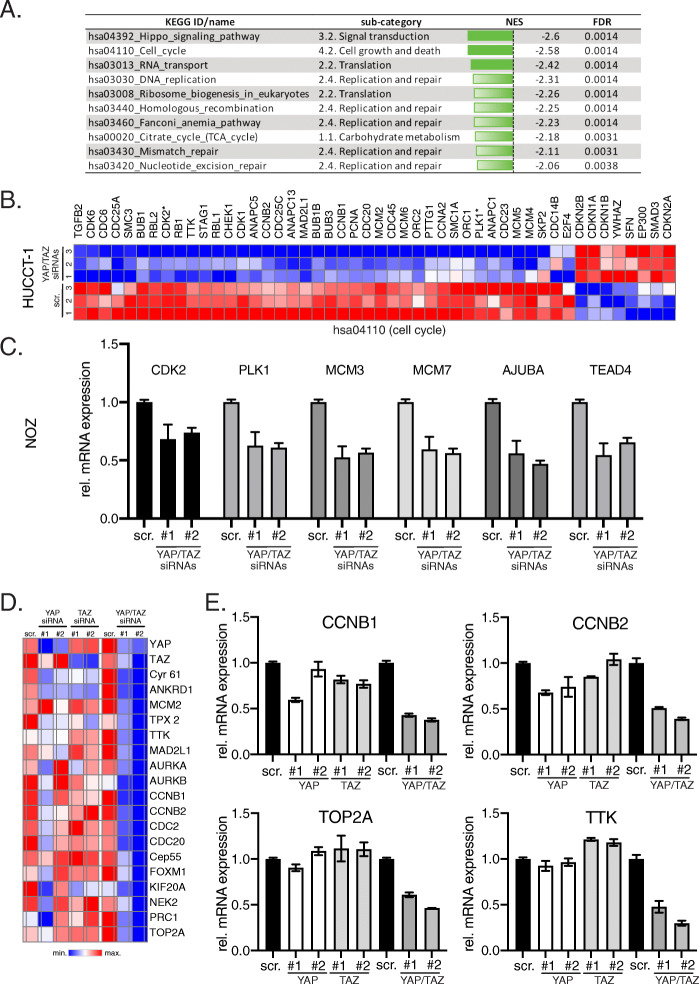
YAP/TAZ control proliferation- and CIN-associated genes in CCA cells. **A** List of identified KEGG pathways and sub-categories with lowest NES after combined YAP/TAZ inhibition in HUCCT-1 cells (FDR ≤ 0.05). **B** Heatmap summarizing significantly regulated genes of ‘cell cycle’ (hsa04110) after combined YAP/TAZ inhibition in HUCCT-1 cells (FDR ≤ 0.05). Genes not significantly regulated are excluded. Genes that were used for independent qPCR confirmation are indicated (*). **C** Independent confirmatory qPCR experiment in NOZ cells with genes identified in the KEGG pathways cell cycle (hsa04110; CDK2, PLK1), DNA replication (hsa03030; MCM3, MCM7) and Hippo signaling pathway (hsa04392; AJUBA, TEAD4). **D** Heatmap summarizing qPCR results from 16 genes that are part of the CIN25 signature in HUCCT-1 cells. CYR61 and ANKRD1 served as positive controls. Two YAP- and TAZ-specific siRNAs were transfected individually and in combination. **E** Exemplary bar graphs for the CIN signature genes CCNB1, CCNB2, TOP2A and TTK in HUCCT-1 cells after YAP/TAZ inhibition

In total, 542 defined genes were significantly and differentially expressed (269 genes upregulated and 273 genes downregulated), which corresponded with several significantly regulated categories after GSEA (Fig. 4a). Among the 10 KEGG IDs with lowest *normalized enrichment scores* (NES), the categories cell cycle (hsa04110; Fig. 4b) and DNA replication (hsa03030, Supplementary Figure S3A) confirmed a direct impact of YAP and TAZ on cell proliferation as already described for hepatocytes and other cell types. Importantly, 5/10 categories with significant NES were also associated with processes involved in DNA repair (DNA replication, homologous recombination, Fanconi anemia pathway, mismatch repair, and nucleotide excision repair), strongly suggesting that aberrant YAP/TAZ expression could be involved in the accumulation of mutations and genomic alterations. Lastly, several genes involved in Hippo pathway signaling were regulated upon YAP/TAZ silencing (NES: − 2.6; *p* ≤ 0.0014), pointing to the existence of feedback mechanisms acting on the Hippo/YAP/TAZ pathway (Supplementary Figure S3A). The regulatory impact of YAP/TAZ on selected candidates associated with cell cycle control (CDK2, PLK1), DNA replication (MCM3, MCM7) and Hippo signaling (AJUBA, TEAD4) was confirmed in different CCA cell lines after combined YAP/TAZ silencing (Fig. 4c, Supplementary Figure S3B), illustrating that these target gene effects were not restricted to HUCCT-1 cells.

Because the transcriptome data suggested that YAP/TAZ silencing may affect the abundance of genomic alterations, we hypothesized that both factors contribute to CIN in CCA. Indeed, YAP activation has been described as inducer of CIN in hepatocyte- and cholangiocyte-derived tumors [3, 24]. This association was illustrated by the presence of a so-called CIN gene signature (CIN25) in independent mouse models with YAP-associated tumor development [3, 18, 24]. Since our expression profiling approach used a YAP/TAZ siRNA combination, we assessed whether YAP or TAZ alone could control the expression of these genes in CCA cells. For this purpose, we analyzed a panel of 16 CIN-associated genes (e.g., AURKA, AURKB, CCNB1, CCNB2, FOXM1) after individual and combined YAP/TAZ silencing using different siRNAs in HUCCT-1 cells [3, 18]. Indeed, qPCR analyses revealed that only the combined inhibition of YAP and TAZ led to a prominent reduction of all investigated CIN signature genes, while the known YAP/TAZ target genes CYR61 and ANKRD1 were already consistently diminished after single YAP or TAZ inhibition (Fig. 4d/e). The strong effects of combined YAP/TAZ silencing on CIN-associated target genes suggested that YAP/TAZ co-expression is of special importance for the induction of CIN and therefore may facilitate a selection advantage for cholangiocyte-derived tumor cells.

In sum, these results illustrate cooperative YAP/TAZ-dependent control of genes that are critical for the induction of CIN in tumorigenesis.

### The presence of YAP-induced CIN genes correlates with poor clinical outcome

The fact that YAP/TAZ cooperate in the regulation of CIN genes was surprising, since a prominent YAP/TAZ co-induction was detectable only in few cases of human CCA samples (Fig. 1). We hypothesized that YAP/TAZ co-expression may provide a growth advantage for distinct CCA cells, which are prone to the development of CIN.

**Fig. 5:**
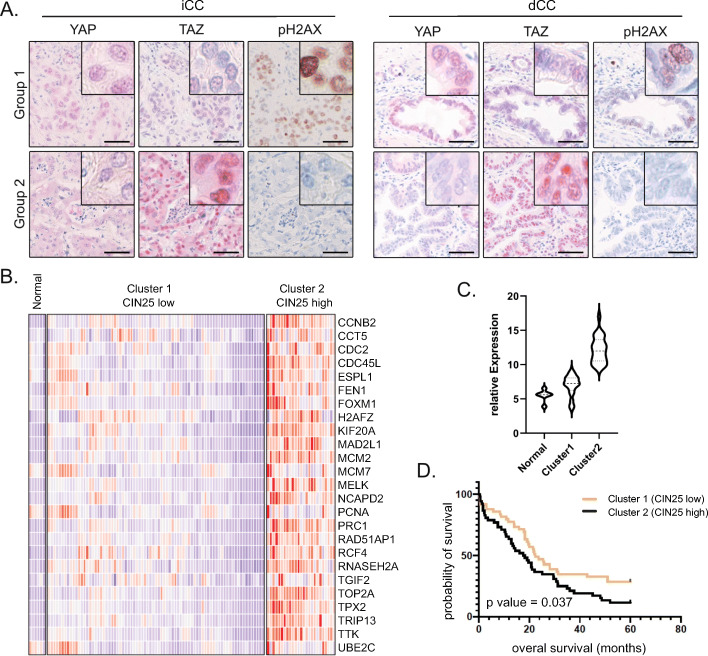
Nuclear YAP enrichment and activity associate with the CIN marker pH2AX and poor clinical outcome. **A** Examples for YAP, TAZ and pH2AX positivity in human iCCA and dCCA cohorts. Bars: 50 μm. **B** Heatmap summarizing the expression of all CIN25 gene signature genes in an iCCA patient cohort. Expression data was divided into 3 groups by using the k-mean clustering algorithm (normal, cluster 1 and cluster 2). **C** Distribution of CIN25 gene signature is illustrated by the CIN gene score in normal bile duct tissue (Normal), Cluster 1 (low CIN gene signature score) and Cluster 2 (high CIN gene signature score). **D** Differences in overall survival of iCCA patients after median clustering of patients based on high or low relative expression of the CIN25 signature. Statistical test: Log-rank (Mantel-Cox)

For this reason, we tested whether YAP or TAZ expression in CCA subtypes was associated with the abundance of phosphorylated H2AX (pH2AX), which is a marker for DNA double strand breaks and genomic instability [25]. IHC stains followed by statistical comparison revealed that for nuclear YAP expression a significant correlation was detected in iCCA and dCCA samples (iCCA: *r* = 0.34, dCCA: *r* = 0.32; *p* ≤ 0.001), while no significant association was observed for TAZ in all CCA subtypes (Fig. 5a). These results would argue for a less pronounced role of TAZ in a tissue context.

To corroborate the clinical relevance of the described YAP-induced CIN25 gene signature, we analyzed an independent cohort of 104 iCCA patients [17]. Indeed, in comparison to normal bile duct tissue, 25% of all patient samples presented a strong enrichment of 25 CIN genes (Fig. 5b). To correlate the presence of CIN genes with patient survival, we calculated a gene signature score that included all CIN25 genes in a balanced manner (Fig. 5c). The results illustrated that the presence of the CIN signature indeed significantly correlated with worse clinical outcome (Fig. 5d).

Together, these results illustrate that a YAP-induced CIN signature is detectable in a subgroup of iCCA patients, which correlates with poor clinical outcome.

## Discussion

Dysregulation of the Hippo signaling pathway and its two transcriptional downstream effectors YAP and TAZ is important for the development of different pathologies in multiple tissues [1, 26]. For the liver it has been demonstrated that YAP and TAZ activation control cellular stemness and influence tumor cell fate as well as genomic integrity. Due to its impact on these tumor-relevant processes, its dysregulation contributes to the development of different tumor types [4]. In this context, subcellular localization and activity of the oncogenes YAP/TAZ are controlled by a central kinase cassette consisting of MST1/2 and LATS1/2 with direct impact on cell biology via the regulation of common YAP/TAZ transcriptional targets [7, 27]. Although YAP and TAZ share most of their transcriptional program, knock-out studies demonstrated different phenotypes in mice, strongly suggesting also exclusive functions of YAP and TAZ [28, 29]. Additionally, YAP and TAZ do not equally contribute to e.g., breast cancer and liver fibrosis in nonalcoholic steatohepatitis [4, 30], further substantiating the idea that both factors must be comparatively investigated under distinct disease conditions. Because a comprehensive analysis of YAP/TAZ expression in CCA and its subtypes (iCCA, pCCA and dCCA) is missing, we decided to characterize the nuclear and cytoplasmic abundance of both factors in recently published cohorts [16].

While no substantial differences between the investigated subtypes regarding nuclear YAP/TAZ enrichment were detected, YAP/TAZ co-expression in only a minority of CCA cases caught our attention. Importantly, all investigated cholangiocyte-derived tumor cell lines showed either a prominent co-expression of both factors or no YAP/TAZ expression, which suggested that the presence of YAP/TAZ could facilitate a growth/selection advantage for these cells. Those CCA cells without YAP/TAZ expression may have developed Hippo/YAP/TAZ-independent mechanisms allowing them to grow outside the tissue context. Another conclusion of our findings is that especially at the tissue level, YAP and TAZ are regulated by distinct molecular mechanisms. This is illustrated by the fact that for up to 49.2% of all CCAs an exclusive nuclear YAP or TAZ expression was detectable. Possible molecular mechanisms for the differential abundance of YAP and TAZ have not been investigated in detail; however, previous results suggested that YAP and TAZ stability could explain this observation [31, 32].

Our results are pointing to an important role of YAP, and potentially TAZ, in the regulation of CIN in CCA cells. We show that YAP not only correlates with positivity of the genomic instability marker pH2AX in CCA tissues but also that YAP and TAZ cooperate in the regulation of the CIN25 gene signature [18]. This conclusion was confirmed by previous findings illustrating that YAP physically interacts with the transcription factor FOXM1 to drive the expression of CIN genes in HCC [3, 23]. Importantly, similar mechanisms for YAP-dependent CIN induction have been described for a syngeneic orthotopic CCA model [24]. Although, to our knowledge, a direct link between TAZ and CIN development has not been described, several lines of evidence for the upstream Hippo kinase cassette further suggest the relevance of the Hippo/YAP/TAZ signaling axis in CIN. For example, inactivation of the serine/threonine kinase LATS1, which directly phosphorylates/inactivates YAP and TAZ, caused CIN and tumor formation [33]. Equally, MST1 - also known as *serine/threonine kinase 4* (STK4) - has been described as critical factor for the maintenance of genomic integrity in lymphoma [34]. If YAP and TAZ equally contribute to the regulation of CIN-relevant genes (as illustrated for the CCA cell lines) or if YAP is a denominator for this phenotype as indicated by the association between nuclear YAP and pH2AX in CCA tissue, should be investigated in future studies.

Although, iCCA, pCCA and dCCA differ regarding risk factors, epidemiology and prognosis [35], no obvious differences for YAP and/or TAZ expression and dynamic shuttling were detectable in our study. However, an important finding is that all investigated subtypes show significant nuclear YAP, TAZ or YAP/TAZ expression to a comparable extent, which qualifies them as eligible for YAP/TAZ-directed therapies. Indeed, first studies already illustrated that Verteporfin, which affects YAP/TEAD and TAZ/TEAD interactions or Thiostrepton, which inhibits FOXM1 could partly abolish YAP-induced effects in vitro and in vivo [3, 24, 36]. In addition, several novel drugs that disturb YAP/TAZ interaction with TEAD are currently investigated. For example, specific targeting of the TEAD lipid pocket disrupts TEAD S-palmitoylation, which reduces its transcriptional activity in a dominant-negative manner [37]. It is therefore tempting to speculate that both CCAs with exclusive nuclear YAP or TAZ accumulation and CCAs with cooperative mutual nuclear YAP/TAZ enrichment could benefit from these innovative treatment strategies in the future.

## Conclusions

Nuclear YAP and TAZ are expressed in all investigated CCA subtypes with comparable frequency. Although, YAP and TAZ are controlled by identical upstream kinases, their co-expression is detectable with lower frequency. This points to the existence of YAP- and TAZ-specific molecular mechanisms that differentially control their subcellular localization (e.g., varying shuttling dynamics from the cytoplasm to the nucleus). YAP/TAZ cooperatively control genes involved in proliferation as well as CIN. Because the presence of YAP/TAZ-induced CIN genes signatures define CCA patients with poor clinical outcome, this group may especially benefit from novel YAP/TAZ-directed therapies, which are currently developed.

## Supplementary Information

**Additional file 1: Figure S1.** YAP and TAZ shuttle in HuH-28 and G415 cells in a cell density-dependent manner

**Additional file 2: Figure S2.** Function of YAP/TAZ expression in HuH-28 and G415 cells

**Additional file 3: Figure S3.** Expression analysis of CCA cell lines after YAP/TAZ inhibition

**Additional file 4: Suppl. Table S1.** iCCA, dCCA and pCCA cohorts

**Additional file 5: Suppl. Table S2.** Primers and antibodies used in this study

## Data Availability

Raw and normalized data were deposited in the Gene Expression Omnibus database (available: http://www.ncbi.nlm.nih.gov/geo/; accession number: GSE172135).

## Abbreviations

CCA: Cholangiocarcinoma
CIN: Chromosomal instability
CTGF: Connective tissue growth factor
CYR61: Cysteine-rich heparin-binding protein 61
dCCA: distal cholangiocarcinoma
eCCA: extrahepatic cholangiocarcinoma
FOXM1: Forkhead box M1
HCC: Hepatocellular carcinoma
iCCA: intrahepatic cholangiocarcinoma
MCM2: Minichromosome maintenance complex component-2
MST1/2: Mammalian STE20-like protein kinase (MST)-1/2
pCCA: perihilar cholangiocarcinoma
pH2AX: phospho-H2A histone family member X
TAZ: WWTR1, WW domain containing transcription regulator 1
TEAD: TEA domain transcription factors
YAP: Yes-associated protein

## References

[1] Zhao B, Tumaneng K, Guan KL (2011). The hippo pathway in organ size control, tissue regeneration and stem cell self-renewal. Nat Cell Biol.

[2] Eisinger-Mathason TS, Mucaj V, Biju KM, Nakazawa MS, Gohil M, Cash TP, Yoon SS, Skuli N, Park KM, Gerecht S (2015). Deregulation of the hippo pathway in soft-tissue sarcoma promotes FOXM1 expression and tumorigenesis. Proc Natl Acad Sci U S A.

[3] Weiler SME, Pinna F, Wolf T, Lutz T, Geldiyev A, Sticht C, Knaub M, Thomann S, Bissinger M, Wan S, Rössler S, Becker D, Gretz N, Lang H, Bergmann F, Ustiyan V, Kalin TV, Singer S, Lee JS, Marquardt JU, Schirmacher P, Kalinichenko VV, Breuhahn K (2017). Induction of chromosome instability by activation of yes-associated protein and Forkhead box M1 in liver Cancer. Gastroenterology.

[4] Zanconato F, Cordenonsi M, Piccolo S (2016). YAP/TAZ at the roots of Cancer. Cancer Cell.

[5] Patel SH, Camargo FD, Yimlamai D (2017). Hippo signaling in the liver regulates organ size, cell fate, and carcinogenesis. Gastroenterology.

[6] Wang H, Wang J, Zhang S, Jia J, Liu X, Zhang J, Wang P, Song X, Che L, Liu K, Ribback S, Cigliano A, Evert M, Wu H, Calvisi DF, Zeng Y, Chen X (2020). Distinct and overlapping roles of hippo effectors YAP and TAZ during human and mouse Hepatocarcinogenesis. Cell Mol Gastroenterol Hepatol.

[7] Weiler SME, Lutz T, Bissinger M, Sticht C, Knaub M, Gretz N, Schirmacher P, Breuhahn K (2020). TAZ target gene ITGAV regulates invasion and feeds back positively on YAP and TAZ in liver cancer cells. Cancer Lett.

[8] Li H, Wolfe A, Septer S, Edwards G, Zhong X, Abdulkarim AB, Ranganathan S, Apte U (2012). Deregulation of hippo kinase signalling in human hepatic malignancies. Liver Int.

[9] Marti P, Stein C, Blumer T, Abraham Y, Dill MT, Pikiolek M, Orsini V, Jurisic G, Megel P, Makowska Z, Agarinis C, Tornillo L, Bouwmeester T, Ruffner H, Bauer A, Parker CN, Schmelzle T, Terracciano LM, Heim MH, Tchorz JS (2015). YAP promotes proliferation, chemoresistance, and angiogenesis in human cholangiocarcinoma through TEAD transcription factors. Hepatology.

[10] Pei T, Li Y, Wang J, Wang H, Liang Y, Shi H, Sun B, Yin D, Sun J, Song R, Pan S, Sun Y, Jiang H, Zheng T, Liu L (2015). YAP is a critical oncogene in human cholangiocarcinoma. Oncotarget.

[11] Sugimachi K, Nishio M, Aishima S, Kuroda Y, Iguchi T, Komatsu H, Hirata H, Sakimura S, Eguchi H, Bekki Y, Takenaka K, Maehara Y, Suzuki A, Mimori K (2017). Altered expression of hippo signaling pathway molecules in intrahepatic cholangiocarcinoma. Oncology.

[12] Wu H, Liu Y, Jiang XW, Li WF, Guo G, Gong JP, Ding X (2016). Clinicopathological and prognostic significance of yes-associated protein expression in hepatocellular carcinoma and hepatic cholangiocarcinoma. Tumour Biol.

[13] Lu L, Finegold MJ, Johnson RL (2018). Hippo pathway coactivators yap and Taz are required to coordinate mammalian liver regeneration. Exp Mol Med.

[14] Calses PC, Crawford JJ, Lill JR, Dey A (2019). Hippo pathway in Cancer: aberrant regulation and therapeutic opportunities. Trends Cancer.

[15] Johnson R, Halder G (2014). The two faces of hippo: targeting the hippo pathway for regenerative medicine and cancer treatment. Nat Rev Drug Discov.

[16] Albrecht T, Rausch M, Rossler S, Albrecht M, Braun JD, Geissler V, Mehrabi A, Vogel MN, Pathil-Warth A, Mechtersheimer G (2019). HER2 gene (ERBB2) amplification is a rare event in non-liver-fluke associated cholangiocarcinogenesis. BMC Cancer.

[17] Andersen JB, Thorgeirsson SS (2012). Genetic profiling of intrahepatic cholangiocarcinoma. Curr Opin Gastroenterol.

[18] Carter SL, Eklund AC, Kohane IS, Harris LN, Szallasi Z (2006). A signature of chromosomal instability inferred from gene expression profiles predicts clinical outcome in multiple human cancers. Nat Genet.

[19] Gu Z, Eils R, Schlesner M (2016). Complex heatmaps reveal patterns and correlations in multidimensional genomic data. Bioinformatics.

[20] Arganda-Carreras I, Kaynig V, Rueden C, Eliceiri KW, Schindelin J, Cardona A, Sebastian SH (2017). Trainable Weka segmentation: a machine learning tool for microscopy pixel classification. Bioinformatics.

[21] Schindelin J, Arganda-Carreras I, Frise E, Kaynig V, Longair M, Pietzsch T, Preibisch S, Rueden C, Saalfeld S, Schmid B, Tinevez JY, White DJ, Hartenstein V, Eliceiri K, Tomancak P, Cardona A (2012). Fiji: an open-source platform for biological-image analysis. Nat Methods.

[22] Halder G, Dupont S, Piccolo S (2012). Transduction of mechanical and cytoskeletal cues by YAP and TAZ. Nat Rev Mol Cell Biol.

[23] Wei T, Weiler SME, Toth M, Sticht C, Lutz T, Thomann S, De La Torre C, Straub B, Merker S, Ruppert T (2019). YAP-dependent induction of UHMK1 supports nuclear enrichment of the oncogene MYBL2 and proliferation in liver cancer cells. Oncogene.

[24] Rizvi S, Fischbach SR, Bronk SF, Hirsova P, Krishnan A, Dhanasekaran R, Smadbeck JB, Smoot RL, Vasmatzis G, Gores GJ (2018). YAP-associated chromosomal instability and cholangiocarcinoma in mice. Oncotarget.

[25] Valdiglesias V, Giunta S, Fenech M, Neri M, Bonassi S (2013). gammaH2AX as a marker of DNA double strand breaks and genomic instability in human population studies. Mutat Res.

[26] Manno G, Filorizzo C, Fanale D, Brando C, Di Lisi D, Lunetta M, Bazan V, Russo A, Novo G (2021). Role of the HIPPO pathway as potential key player in the cross talk between oncology and cardiology. Crit Rev Oncol Hematol.

[27] Zanconato F, Forcato M, Battilana G, Azzolin L, Quaranta E, Bodega B, Rosato A, Bicciato S, Cordenonsi M, Piccolo S (2015). Genome-wide association between YAP/TAZ/TEAD and AP-1 at enhancers drives oncogenic growth. Nat Cell Biol.

[28] Makita R, Uchijima Y, Nishiyama K, Amano T, Chen Q, Takeuchi T, Mitani A, Nagase T, Yatomi Y, Aburatani H, Nakagawa O, Small EV, Cobo-Stark P, Igarashi P, Murakami M, Tominaga J, Sato T, Asano T, Kurihara Y, Kurihara H (2008). Multiple renal cysts, urinary concentration defects, and pulmonary emphysematous changes in mice lacking TAZ. Am J Physiol Renal Physiol.

[29] Morin-Kensicki EM, Boone BN, Howell M, Stonebraker JR, Teed J, Alb JG, Magnuson TR, O'Neal W, Milgram SL (2006). Defects in yolk sac vasculogenesis, chorioallantoic fusion, and embryonic axis elongation in mice with targeted disruption of Yap65. Mol Cell Biol.

[30] Wang X, Zheng Z, Caviglia JM, Corey KE, Herfel TM, Cai B, Masia R, Chung RT, Lefkowitch JH, Schwabe RF, Tabas I (2016). Hepatocyte TAZ/WWTR1 promotes inflammation and fibrosis in nonalcoholic steatohepatitis. Cell Metab.

[31] Azzolin L, Zanconato F, Bresolin S, Forcato M, Basso G, Bicciato S, Cordenonsi M, Piccolo S (2012). Role of TAZ as mediator of Wnt signaling. Cell.

[32] Liu CY, Zha ZY, Zhou X, Zhang H, Huang W, Zhao D, Li T, Chan SW, Lim CJ, Hong W, Zhao S, Xiong Y, Lei QY, Guan KL (2010). The hippo tumor pathway promotes TAZ degradation by phosphorylating a phosphodegron and recruiting the SCF {beta}-TrCP E3 ligase. J Biol Chem.

[33] Yabuta N, Mukai S, Okamoto A, Okuzaki D, Suzuki H, Torigata K, Yoshida K, Okada N, Miura D, Ito A, Ikawa M, Okabe M, Nojima H (2013). N-terminal truncation of Lats1 causes abnormal cell growth control and chromosomal instability. J Cell Sci.

[34] Kim TS, Lee DH, Kim SK, Shin SY, Seo EJ, Lim DS (2012). Mammalian sterile 20-like kinase 1 suppresses lymphoma development by promoting faithful chromosome segregation. Cancer Res.

[35] Banales JM, Marin JJG, Lamarca A, Rodrigues PM, Khan SA, Roberts LR, Cardinale V, Carpino G, Andersen JB, Braconi C, Calvisi DF, Perugorria MJ, Fabris L, Boulter L, Macias RIR, Gaudio E, Alvaro D, Gradilone SA, Strazzabosco M, Marzioni M, Coulouarn C, Fouassier L, Raggi C, Invernizzi P, Mertens JC, Moncsek A, Rizvi S, Heimbach J, Koerkamp BG, Bruix J, Forner A, Bridgewater J, Valle JW, Gores GJ (2020). Cholangiocarcinoma 2020: the next horizon in mechanisms and management. Nat Rev Gastroenterol Hepatol.

[36] Camargo FD, Gokhale S, Johnnidis JB, Fu D, Bell GW, Jaenisch R, Brummelkamp TR (2007). YAP1 increases organ size and expands undifferentiated progenitor cells. Curr Biol.

[37] Holden JK, Crawford JJ, Noland CL, Schmidt S, Zbieg JR, Lacap JA, Zang R, Miller GM, Zhang Y, Beroza P, Reja R, Lee W, Tom JYK, Fong R, Steffek M, Clausen S, Hagenbeek TJ, Hu T, Zhou Z, Shen HC, Cunningham CN (2020). Small molecule dysregulation of TEAD Lipidation induces a dominant-negative inhibition of hippo pathway signaling. Cell Rep.

