# Supplementary figures and images for: Co-expression of YAP and TAZ associates with chromosomal instability in human cholangiocarcinoma
Source: BMC Cancer. 2021 Oct 6;21:1079. doi: 10.1186/s12885-021-08794-5 (PMC8496054; doi:10.1186/s12885-021-08794-5)

Suppl. Figure S1

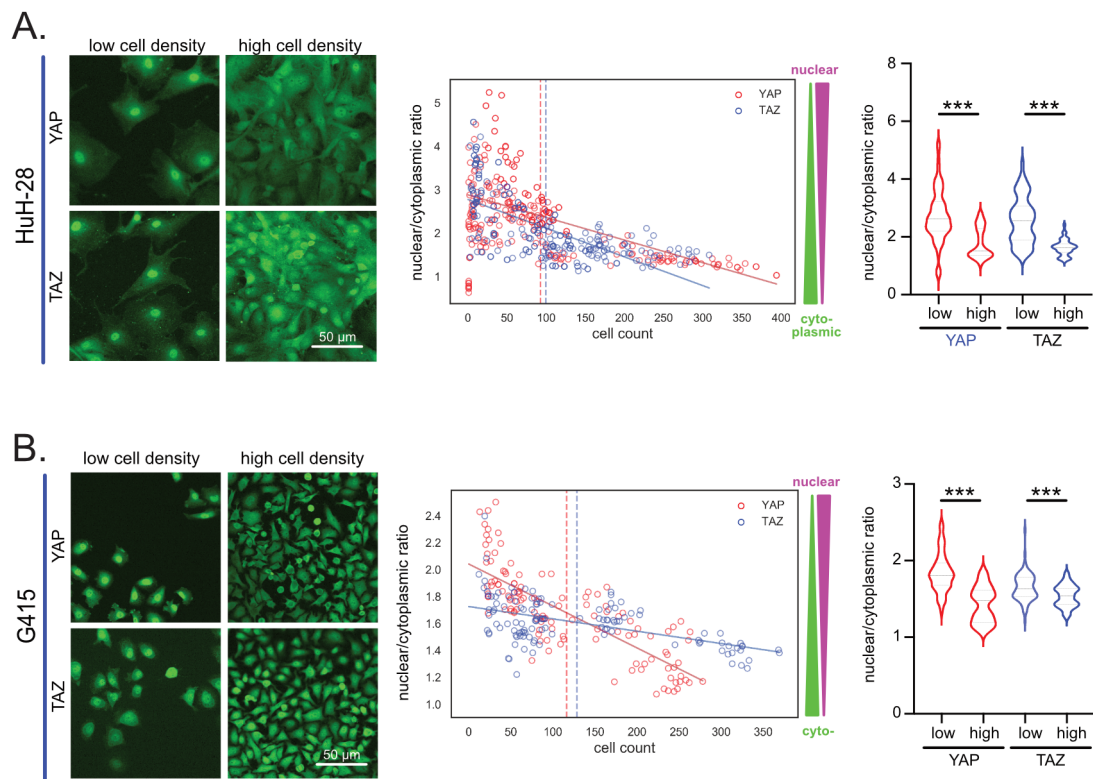

Supplement: Supplementary file 1 — Additional file 1: Figure S1. YAP and TAZ shuttle in HuH-28 and G415 cells in a cell density-dependent manner [file 12885_2021_8794_MOESM1_ESM.pdf]

Supplementary Figure 2

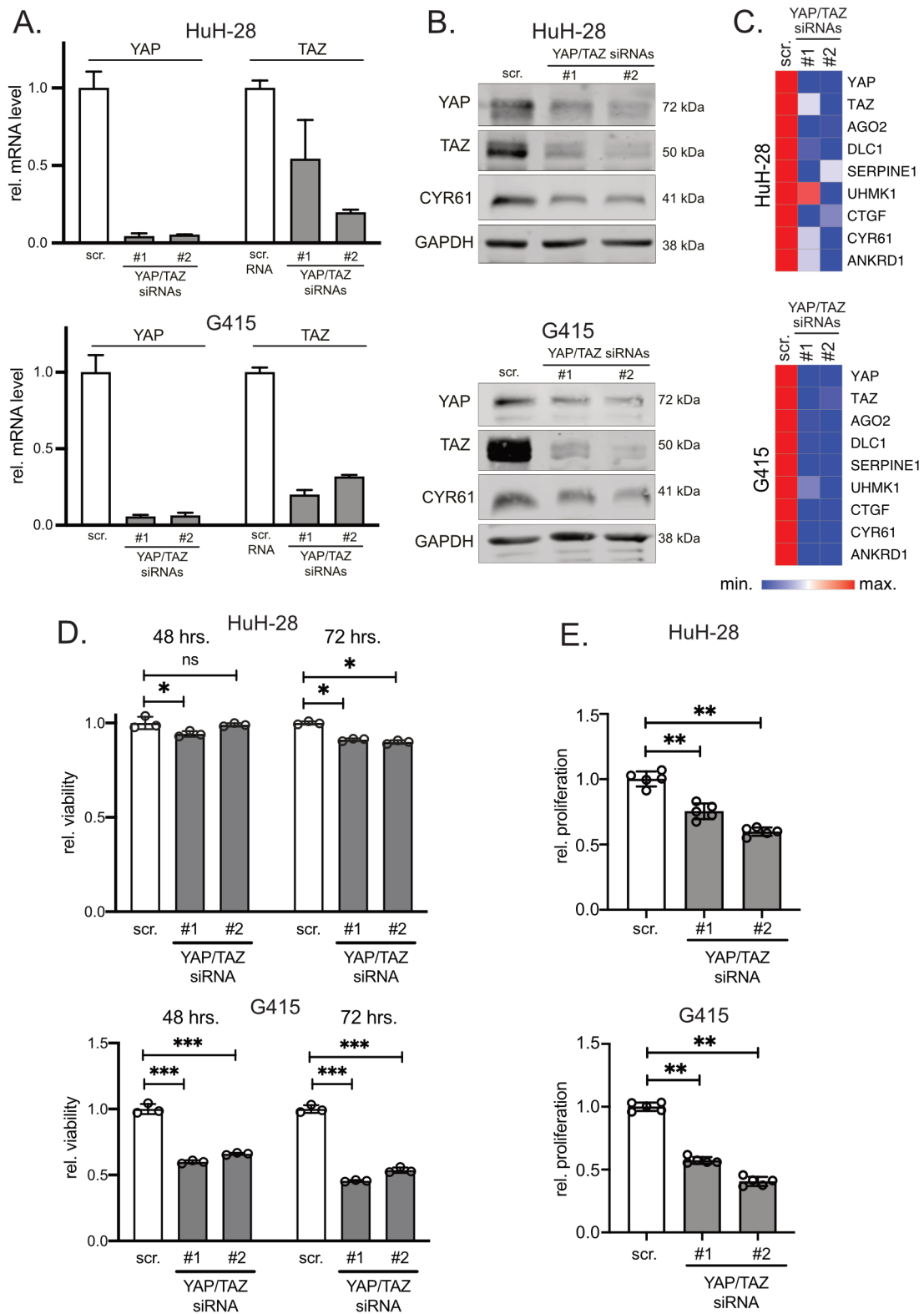

Supplement: Supplementary file 2 — Additional file 2: Figure S2. Function of YAP/TAZ expression in HuH-28 and G415 cells [file 12885_2021_8794_MOESM2_ESM.pdf]

A.

Suppl. Figure S3

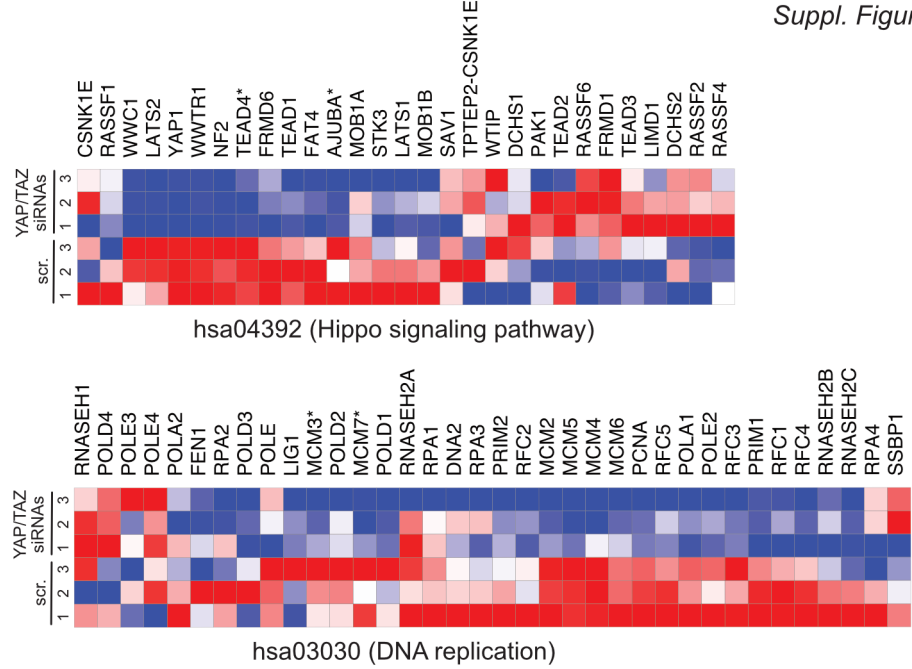

B.

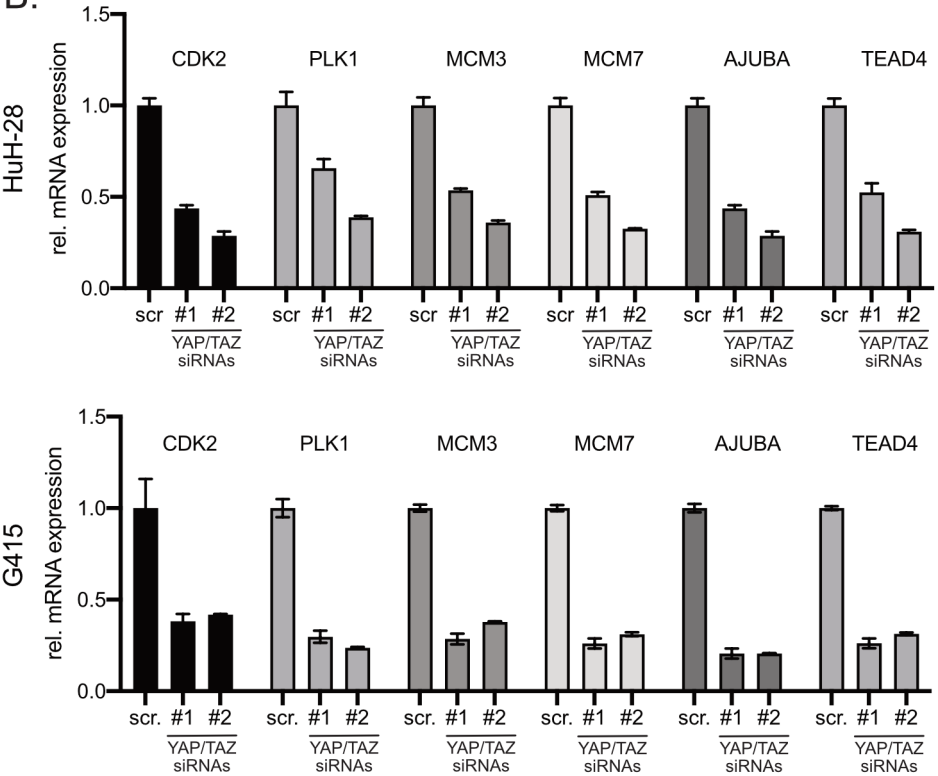

Supplement: Supplementary file 3 — Additional file 3: Figure S3. Expression analysis of CCA cell lines after YAP/TAZ inhibition [file 12885_2021_8794_MOESM3_ESM.pdf]
