# Supplementary material for: Co-expression of YAP and TAZ associates with chromosomal instability in human cholangiocarcinoma
Source: BMC Cancer. 2021 Oct 6;21:1079. doi: 10.1186/s12885-021-08794-5 (PMC8496054; doi:10.1186/s12885-021-08794-5)
Supplement: Supplementary file 4 — Additional file 4: Suppl. Table S1. iCCA, dCCA and pCCA cohorts [file 12885_2021_8794_MOESM4_ESM.docx]

iCCA

| **Clinicopathological Features** | **No. of patients (Total No. = 152)** |
| --- | --- |
| Age (yr)  Mean 61,2  Minimum 30,8  Maximum 86,2 |  |
| Sex  Men  Women | 55,9% (85/152)  44,1% (69/152) |
| T status  T1a  T1b  T2  T3  T4 | 10,5% (16/152)  2,0% (3/152)  59,9% (91/152)  17,7% (27/152)  9,9% (15/152) |
| N status  Nx  N0  N1 | 46,7% (71/152)  30,3% (46/152)  23,0% (35/152) |
| M status  M0  M1 | 96,7% (147/152)  3,3% (5/152) |
| Grading  G1  G2  G3  G4 | 5,9% (9/152)  64,5% (98/152)  27,6% (42/152)  2,0% (3/152) |
| UICC stage  IA  IB  II  IIIA  IIIB  IV  Overal survival (yr)  Mean 2,5  Minimum 0,0  Maximum 11,5  Subtype  Small duct  Large duct | 6,1% (5/82)  0% (0/82)  36,6% (30/82)  9,7% (8/82)  41,5% (34/82)  6,1% (5/82)  60% (91/152)  40% (61/152) |
|  |  |

pCCA

| **Clinicopathological Features** | **No. of patients (Total No. = 155)** |
| --- | --- |
| Age (yr)  Mean 65,2  Minimum 24,4  Maximum 84,3 |  |
| Sex  Men  Women | 67,7% (105/155)  32,3% (50/155) |
| T status  T1  T2a  T2b  T3  T4 | 1,3% (2/155)  27,7% (43/155)  51,0% (79/155)  11,6% (18/155)  8,4% (13/155) |
| N status  Nx  N0  N1 | 16,1% (25/155)  49,7% (77/155)  34,2% (53/155) |
| M status  M0  M1 | 96,1% (149/155)  3,9% (6/155) |
| Grading  G1  G2  G3  G4 | 5,2% (8/155)  73,5% (114/155)  21,3% (33/155)  0% (0/155) |
| UICC stage  I  II  III  IV  Overal survival (yr)  Mean 1,9  Minimum 0,0  Maximum 13,6 | 1,5% (2/131)  48,1% (63/131)  45,8% (60/131)  4,6% (6/131) |

dCCA

| **Clinicopathological Features** | **No. of patients (Total No. = 126)** |
| --- | --- |
| Age (yr)  Mean 68,1  Minimum 19,3  Maximum 88,2 |  |
| Sex  Men  Women | 72,2% (91/126)  27,8% (35/126) |
| T status  T1  T2  T3  T4 | 4% (5/125)  19,2% (24/125)  71,2% (89/125)  5,6% (7/125) |
| N status  Nx  N0  N1 | 1,6% (2/126)  34,1% (43/126)  64,3% (81/126) |
| M status  M0  M1 | 94,4% (119/126)  5,6% (7/126) |
| Grading  G1  G2  G3  G4 | 0,8% (1/126)  66,7% (84/126)  32,5% (41/126)  0% (0/126) |
| UICC stage  I  II  III  IV  Overal survival (yr)  Mean 2,4  Minimum 0,0  Maximum 12,9 | 5,6% (7/125)  88% (100/125)  0,8% (1/125)  5,6% (7/125) |
