# Supplementary material for: Co-expression of YAP and TAZ associates with chromosomal instability in human cholangiocarcinoma
Source: BMC Cancer. 2021 Oct 6;21:1079. doi: 10.1186/s12885-021-08794-5 (PMC8496054; doi:10.1186/s12885-021-08794-5)
Supplement: Supplementary file 5 — Additional file 5: Suppl. Table S2. Primers and antibodies used in this study [file 12885_2021_8794_MOESM5_ESM.docx]

**Supplementary Table S2: List of used antibodies and primers**

| Primary Antibodies (clone) | Source/Company | Identifier |
| --- | --- | --- |
| Actin (C4) | MP Biomedicals (California, USA) | #8691002; RRID: AB_2335304 |
| Cyr61 (D4H5D) | Cell Signaling Technology (Danvers, USA) | #14479; RRID: AB_ 2798492 |
| GAPDH | Merck Millipore (Burlington, USA) | #AB2302; RRID: AB_ 10615768 |
| Ki-67 (MIB-1) | Dako (Hamburg, Germany) | M7240; RRID: AB_2142367 |
| MCM2 (D7G11) | Cell Signaling Technology | #3619; RRID: AB_2142137 |
| pH2AX | Cell Signaling Technology | #9718, RRID: AB_2118009 |
| TAZ for IHC | Abcam (Cambridge, UK) | ab110239, RRID: AB_2889399 |
| TAZ for WB | Cell Signaling Technology | #4883; RRID: AB_1904158 |
| TAZ for IF | Atlas Antibodies (Stockholm, Sweden) | HPA007415; RRID: AB_1080602 |
| YAP for IHC | Cell Signaling Technology | #4912, RRID: AB_2218911 |
| YAP for WB | Cell Signaling Technology | #4912; RRID: AB_2218911 |
| YAP for IF | Bethyl Laboratories (Montgomery, USA) | IHC-00665; RRID: AB_10953166 |

IHC - immunohistochemistry, WB - western immunoblotting, IF - immunofluorescence

| Secondary Antibodies | Source/Company | Identifier |
| --- | --- | --- |
| IRDye 680 LT anti-chicken IgG | Li-Cor Biosciences | #926-68028 |
| IRDye 680 LT anti-mouse IgG | Li-Cor Biosciences | #926-68022 |
| IRDye 800 C anti-rabbit IgG | Li-Cor Biosciences | #926-32213 |

| Primers for qPCR (h) | Genbank ID | Sequence (5'-3') |
| --- | --- | --- |
| AGO2 | NM_ 001164623.3 | for: CATCCCAGCGCCAGCATACT |
|  |  | rev: GGCTTCCTTCAGCACTGTCATG |
| AJUBA | NM_032876 | for: GACATCGTGAGGGTGATATCC |
|  |  | rev: CATCCAGAGGGAAACAGCAG |
| ANKRD1 | NM_014391 | for: AGTAGAGGAACTGGTCACTGG |
|  |  | rev: TGGGCTAGAAGTGTCTTCAGAT |
| AURKA | NM_198433.1 | for: CAGTACATGCTCCATCTTCC |
|  |  | rev: CCGACCTTCAATCATTTCAGG |
| AURKB | NM_001256834 | for: ACTTCGGCTGGTCTGTGCAT |
|  |  | rev: GTGCCACACATTGTCTTCCTC |
| B2M | NM_004048 | for: CACGTCATCCAGCAGAGAAT |
|  |  | rev: TGCTGCTTACATGTCTCGAT |
| CCNB1 | NM_031966 | for: GCTGATCCAAACCTTTGTAGTG |
|  |  | rev: GGCTCTCATGTTTCCAGTGAC |
| CCNB2 | NM_004701 | for: GCGTTGGCATTATGGATCGA |
|  |  | rev: CAAGAGCAGAGCAGTAATCC |
| CDC2 | NM_001786 | for: GTGAAGAGGAAGGGGTTCCTAG |
|  |  | rev: TCCTGCATAAGCACATCCTG |
| CDC20 | NM_001255 | for: AGTGCCGTGGATGCCCATT |
|  |  | rev: GGAGACCAGAGGATGGAGCA |
| CEP55 | NM_018131 | for: CCGAAGAGCTCTTATCTCAG |
|  |  | rev: GTTCCAACAGAGCTACCCTTG |
| CTGF | NM_001901 | for: CCAAGGACCAAACCGTGG |
|  |  | rev: CTGCAGGAGGCGTTGTCAT |
| CDC2 | NM_001786 | for: GTGAAGAGGAAGGGGTTCCTAG |
|  |  | rev: TCCTGCATAAGCACATCCTG |
| Cyr61 | NM_010516 | for: GATCTGTGAAGTGCGTCCTTGTGG |
|  |  | rev: GACACTGGAGCATCCTGCATAAG |
| DLC1 | NM_182643 | for: GATCCCGGAAAGAAGGGATTC |
|  |  | rev: GAAACTGTGCCATCTCAGTC |
| FOXM1 | NM_202003 | for: ATAGCAAGCGAGTCCGCATT |
|  |  | rev: TTCCTCCCCAGGCTGGATTT |
| KIF20A | NM_005733 | for: GACCACTTGTGATGACATCT |
|  |  | rev: AGTTCAGCCAGAGTCTGGTC |
| MAD2L1 | NM_002358.3 | for: CAGACAGATCACAGCTACGG |
|  |  | rev: CCCATTTTTCAGGTACAACCA |
| MCM2 | NM_004526 | for: CATCAGCGACATGTGCAAAG |
|  |  | rev: GTTCACCACCAGGCTCTCAC |
| MCM3 | NM_ 002388.6 | for: TGTGTGGAGGGCATTGTCACT |
|  |  | rev: CCACCAGGGTGGTGAGATCAG |
| MCM7 | NM_005916.5 | for: AGTGCCAAACCAACCGCTCA |
|  |  | rev: GGCTGGGCAATTCTTGTGTTCT |
| NEK2 | NM_002497 | for: ACCATTGGCACAGGCTCCTA |
|  |  | rev: GGAGCCATAGTCAAGTTCTTTCC |
| PLK1 | NM_005030 | for: TTCGATTGCTCCCAGCAGC |
|  |  | rev: GCTGCCCAGCTTTGAGCAAAG |
| PRC1 | NM_003981 | for: CCAGGAGCAGAGACAAGCTT |
|  |  | rev: CAATCTCAGCATCGTGGAGC |
| RPL41 | NM_001035267 | for: AAACCTCTGCGCCATGAGAG |
|  |  | rev: AGCGTCTGGCATTCCATGTT |
| SERPINE1 | NM_008871 | for: GACTTCTCAGAAGTGGAAAGAGCC |
|  |  | rev: CTGAAGTAGAGGGCATTCACCAGC |
| TAZ | NM_015472.4 | for: CAGAGAATCCAGATGGAGAG |
|  |  | rev: GTTGACAGCAGCCTGAACTG |
| TEAD4 | NM_201443.2 | for: TGGAGTTCTCTGCCTTCCTG |
|  |  | rev: GGACTGGCCAATGTGCACGA |
| TOP2A | NM_001067 | for: CCACGATACATCTTTACAATGCTC |
|  |  | rev: CTCAACACGCTGGTTGTCATC |
| TPX2 | NM_012112.4 | for: GAACTACGAAAGCATCCTTCATCTCC |
|  |  | rev: CCTTGGGACAGGTTGAAAGGC |
| TTK | NM_003321 | for: CTATTTGTAAGACACCAAGCAGC |
|  |  | rev: GGTGTTGACAACTGACAAGCAG |
| UHMK1 | NM_175866 | for: GTTGCAGGGTCACAGAAACAT |
|  |  | rev: GGAGAAAAGTGGATTGTAAACAC |
| YAP | NM_006106 | for: CCTGCGTAGCCAGTTACCAA |
|  |  | rev: CCATCTCATCCACACTGTTC |
